# Good Practices for Observational Studies of Maternal Weight and Weight Gain in Pregnancy

**DOI:** 10.1111/ppe.12439

**Published:** 2018-01-18

**Authors:** Jennifer A. Hutcheon, Lisa M. Bodnar

**Affiliations:** ^1^ Department of Obstetrics & Gynaecology University of British Columbia Vancouver BC Canada; ^2^ Departments of Epidemiology and of Obstetrics, Gynecology, and Reproductive Sciences Graduate School of Public Health and School of Medicine University of Pittsburgh Pittsburgh PA

The worldwide obesity epidemic has heightened the importance of understanding how a woman's weight during her reproductive years influences health outcomes in the perinatal period and beyond. In a 2009 report, the National Academy of Sciences/Institute of Medicine Committee (IOM) to Reexamine Pregnancy Weight Gain Guidelines outlined a research agenda to fill the most pressing knowledge gaps.^1^ This agenda, combined with increased availability of data on maternal height, pre‐pregnancy weight, and delivery weight from the 2003 revised US National Vital Statistics System birth records,^2^ has promoted an increase in research this field. Yet, observational studies of maternal obesity and gestational weight gain seeking to understand their causal effects present several distinct methodological challenges that, if not properly accounted for, can bias our understanding of the causal impact of these exposures.

The objective of this commentary was to describe what we view as good practices for characterising and analysing data on maternal weight and weight gain in pregnancy when viewed as study exposures. We discuss the relative merits of methods to account for the natural dependency between gestational weight gain and gestational duration, approaches to analyse single vs serial weight gain measurements, as well as strategies for reducing bias by accounting for temporality, minimising confounding, and quantifying the contribution of measurement error. In the Box 1, we provide a summary of key considerations for the design and interpretation of studies in this field.

**Good practices in studies of pre‐pregnancy weight and weight gain**

- Account for the natural correlation between total pregnancy weight gain and gestational duration by controlling for gestational age as a covariate (for outcomes where births at that gestational age is the correct risk calculation denominator), or standardising weight‐gain‐for‐gestational age using pregnancy weight gain‐for‐gestational‐age charts.
- Minimise the potential for reverse causation when studying outcomes diagnosed before delivery such as preeclampsia or gestational diabetes by restricting weight measurements to those taken before diagnosis or disease onset.
- Use directed acyclic graphs to develop the study's analytic plan, including variable selection for multivariable models.
- Present risk ratios, risk differences, or predicted risks rather than odd ratios for common outcomes such as caesarean delivery or excess post‐partum weight retention.
- Investigate effect measure modification of the pregnancy weight gain‐adverse outcome association by pre‐pregnancy BMI by conducting BMI category‐specific analyses or including an interaction term between pre‐pregnancy BMI and pregnancy weight gain.
- Use a flexible approach to model gestational weight gain or pre‐pregnancy BMI (such as fractional polynomials or restricted cubic splines) to examine U‐ or J‐shaped associations with adverse health outcomes.
- Evaluate the potential magnitude of bias introduced by measurement error in self‐reported weight using quantitative bias analysis.

## Characterising gestational weight gain using a single measurement

Researchers often assess the association between total pregnancy weight gain (ie weight at delivery minus pre‐pregnancy weight) and adverse maternal and newborn health outcomes. The characterisation of gestational weight gain through this single measure is driven largely by data availability: delivery weight is commonly abstracted into large, population‐based perinatal databases and registries, whereas weights from antenatal visits are not. When only a single measure of gestational weight gain is analysed, it is critical to account for the fact that a woman's opportunity to gain weight increases the longer she remains pregnant.^1^ Ignoring the correlation between total pregnancy weight gain and gestational duration will introduce a spurious association between low weight gain and any gestational age‐dependent outcome, including stillbirth, neonatal death, preterm birth, or preeclampsia.^3^ This problem remains when total pregnancy weight gain is categorised in relation to current IOM pregnancy weight gain guidelines (ie, below, within, or above the recommended ranges for term deliveries).^4^ Below, we summarise the merits of different approaches that researchers have used to overcome this problem. Later, we describe approaches for the analysis of serial weight gain.

### Covariate adjustment for gestational age

The simplest approach is to use total gestational weight gain as the main study exposure and adjust for gestational age as a covariate in a multivariable regression model.^5^ The primary advantage of this strategy is its simplicity and interpretability. For outcomes where the number of livebirths at a given gestational age is the correct denominator, such as neonatal mortality, covariate adjustment for gestational age is a reasonable strategy. For such studies, researchers should nevertheless be alert to the issue of collider bias, as adjustment for gestational age could open up back door pathways through which confounders of the exposure‐mediator or mediator outcome relationships could bias estimates.^6^ However, this approach is problematic for many other research questions. Most obviously, it is not possible to adjust for gestational age when the outcome of interest is preterm birth (which is defined by gestational age). However, it is also inappropriate to adjust for gestational age when studying any adverse event that a woman is only at risk of while she remains pregnant, including as stillbirth, preeclampsia, labour induction, or placental abruption. To calculate the risk of these outcomes, the correct denominator is the number of ongoing pregnancies at a given gestational age (rather than the number of births at that gestational age).^7^, ^8^ Adjustment for gestational age in a regression model would have the undesired effect of changing the denominator from ‘ongoing pregnancies’ to ‘other births in that week of gestation’. For these research questions, alternative approaches are needed that account for gestational duration directly in the measure of pregnancy weight gain.

### Rate of weight gain

**Figure 1:**
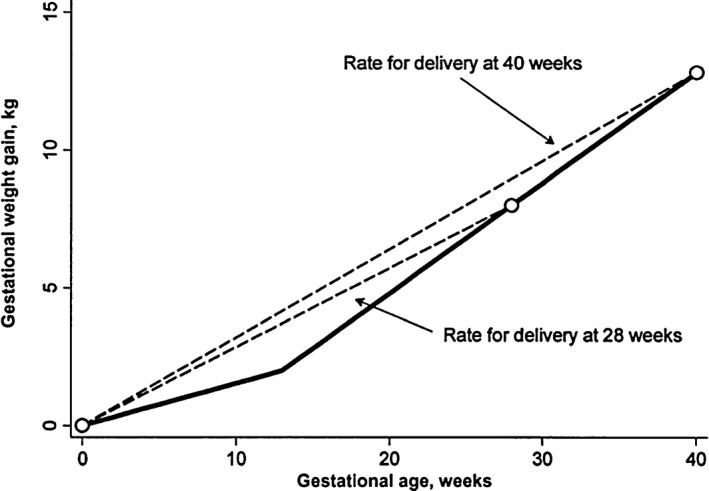
Relationship between gestational age at delivery and average rate of gestational weight gain [*Paediatric and Perinatal Epidemiology* 2012; 26:109–116 Reprinted with permission]

Total pregnancy weight gain can be divided by gestational length to obtain an average weekly rate of weight gain. While this approach considerably reduces the correlation between pregnancy weight gain and gestational duration, it does not fully remove it.^3^, ^9^ It makes the incorrect assumption that pregnancy weight gain is linear across gestation (when averaging across the total number of weeks’ gestation) or linear from 14 weeks’ (when averaging across the number of weeks’ gestation in the second and third trimester). In actuality, the rate of weight gain is slower in the first trimester, and has an inflection point that varies according to pre‐pregnancy body mass index (BMI).^10^, ^11^ Pregnancies with shorter gestations will have rates of weight gain that are artificially low (Figure 1).^3^ Accordingly, studies that use this measure will observe a spurious association between low weight gain and adverse outcomes. Similar biases result from use of the IOM adequacy ratio, which is the ratio of a woman's observed (total) gestational weight gain to the gestational weight gain recommended by the IOM guidelines based on her gestational age at delivery.

### Weight‐gain‐for‐gestational‐age z‐scores

Pregnancy weight‐gain‐for‐gestational‐age charts are a newer tool for isolating total weight gain from gestational duration.^12^, ^13^, ^14^, ^15^ Similar to the estimated fetal weight‐for‐gestational age charts used to assess fetal growth,^16^, ^17^, ^18^, ^19^ the charts describe the mean, standard deviation, and select percentiles of weight gain across gestation in a cohort of women with uncomplicated term pregnancies. Separate charts are available for each pre‐pregnancy BMI category. These reference values can then be used to express a woman's total pregnancy weight gain in relation to that of her peers at a similar gestational age as a standardised pregnancy weight gain *z*‐score. The weight gain z‐score is calculated as: (observed total weight gain − mean week‐specific weight gain)/standard deviation of week‐specific weight gain, with week‐specific means and standard deviations obtained from the reference chart. For example, consider a normal‐weight woman who delivered at 36 weeks with a total pregnancy weight gain of 13 kg. According to the INTERGROWTH weight‐gain‐for‐gestational‐age chart, the median total weight gain at 36 weeks is 11.6 kg. Her weight gain‐for‐gestational‐age *z*‐score would be calculated as: ([ln(13+c)]−3.0122582)/0.19017637, where c is a constant of 8.5, and 3.0122582 and 0.19017637 are the log‐transformed means and standard deviations of weight gain at 36 weeks on the reference chart. The resulting *z*‐score of +0.29 confirms that this woman's weight gain is above average, given her gestational duration. By design, the z‐scores are uncorrelated with gestational age, so can be used to disentangle the effects of weight gain from gestational duration.

Interpretation of weight gain *z*‐scores may be less intuitive than absolute weight gain, so conversion of the *z*‐scores back to kilograms (eg the number of kilograms corresponding to 0.2 weight gain *z*‐score at 40 weeks’) may be useful for presenting and discussing study findings.^20^ The charts also require an assumption that the patterns of weight gain in the study population are similar to those of the population used to derive the chart. The recent publication of a weight gain chart from a global population (based on the INTERGROWTH 21st cohort) should help increase generalisability and comparability of studies across different jurisdictions.^14^

### Time‐varying exposure

The correlation between pregnancy weight gain and gestational duration can also be addressed through the use of time‐to‐event analysis in which weight gain is treated as a time‐varying exposure.^21^ In this approach, a woman's weight at the time of an adverse event is compared to the weights of all other women with ongoing pregnancies at that gestational age who had not experienced an adverse event. This comparison is repeated at the time of each adverse event in the cohort, and a summary estimate of the extent to which the instantaneous risk of an adverse event differs according to pregnancy weight gain status is calculated using Cox‐proportional hazards regression or other survival analysis approaches. While theoretically sound, questions remain about its practical implementation. The approach requires daily weight gain measurements throughout gestation, which are rarely available. Researchers must therefore estimate these measurements by interpolating between available data points, but the best strategy for doing so (as well as the minimum number of measurements and/or timing of measurements) is unclear. Approaches are needed to account for the additional statistical uncertainty introduced by combining estimated measurements with actual measurements in a regression model. Finally, the biological interpretability of the resulting hazards ratio (ie the meaning of the relative increase or decrease in risk per 1 kg difference in pregnancy weight gain) is limited unless more advanced specifications (such as non‐proportionality of hazards) allow the effects of a 1 kg difference on risks to differ according to gestational stage.

## Characterising gestational weight gain using serial measurements

Summarising pregnancy weight gain through a single measure ignores the pattern in which the weight was gained. Some research suggests that certain periods of pregnancy may be more sensitive to the effects of high or low weight gain. For example, excessive gestational weight gain in the first trimester, but not the second or third trimesters, has been associated with childhood obesity at age 5.^22^, ^23^, ^24^, ^25^ This may explain why randomised trials intervening on pregnancy weight gain in mid‐late pregnancy have not always demonstrated reductions in adverse birth outcomes despite reductions in total weight gain.^26^ With the growing availability of electronic antenatal medical records containing serial weight measurements, analyses of longitudinal weight gain patterns at the population level are increasingly possible and may provide new insights into the consequences of pregnancy weight gain on health. Below, we summarise methods that can be used to characterise weight gain patterns throughout gestation. As each of these approaches has some limitations (in ease of interpretation, translation of findings to clinical care, or analytic issues), the best choice will likely differ according to each study's specific research question. Future research to address the shortcomings of existing approaches would be valuable.

### Trimester‐specific rate of weight gain

A common and straightforward approach to classify serial weight gain measurements is to calculate the rate of pregnancy weight gain within each trimester.^24^, ^27^ From a methodological perspective, this approach is similar to the calculation of rate of weight gain based on total pregnancy weight gain discussed above. While it imposes an assumption that weight gain is linear within the time period, this assumption is more reasonable within the shorter time periods of a trimester. However, the approach does not incorporate information on subsequent or previous weight gain patterns. For example, the health consequences of a high rate of second‐trimester weight gain may differ if it was a continuation of high weight gain in the first trimester vs compensatory gain to account for low weight gain in the first trimester linked with nausea and vomiting. To describe the synergy of weight gain patterns, variables indicating each possible permutation of first, second, and third trimester gain must be created, which will quickly run into concerns with sparse data. As a result, approaches that consider all weight gain measurements simultaneously may be preferable when investigating the role of trajectory.

### Area under the curve

Repeated weight gain measurements can be summarised by calculating an area under the curve (AUC) for pregnancy weight gain.^11^ The area under the curve is calculated by plotting each of a woman's serial weight measurements against gestational age, such that trapezoids can be drawn between successive weight measurements (ie drawing a perpendicular line from each of 2 weight measurements to the associated gestational ages at which they were collected, and connecting these lines by joining the successive weight measurements and successive gestational age values). Trapezoids are created to include all weight measurements throughout pregnancy. The area of each trapezoid is calculated, and the area under the curve is obtained by summing the area from all trapezoids.

In this approach, weight gained in early pregnancy will produce a higher AUC than a woman with the same total pregnancy weight gain but who gained more weight in late pregnancy. An advantage of the AUC is that it provides a simple, easy‐to‐calculate method for synthesising multiple weight gain measurements into a single summary measure (interpreted as “pound‐days”). However, the measure is dependent on gestational duration, so may be less useful for studying adverse outcomes that occur at systematically younger gestational ages.

### Random effects models

Serial pregnancy weight gain measurements can also be modelled as a function of gestational age using a multilevel random effects model that produces smoothed individual‐level estimates of maternal weight gain at select times across pregnancy.^28^ The multilevel model accounts for the correlation between a woman's repeated weight measurements, and allows each woman's trajectory to vary about the population average. Gestational age should be modelled using restricted cubic splines or other non‐linear approaches, allowing the pattern of weight gain to follow a flexible, curvilinear pattern across gestation. The individual‐level estimates from different gestational age periods can then be linked with health outcomes as independent variables in a linear or logistic regression model.

By using estimated, rather than actual, weight gain measurements in the final analytic model, the approach imposes a number of assumptions, such as that women's weight gain trajectories follow a normal distribution about the population average trajectory. Furthermore, the approach makes it challenging to disentangle the effect of weight gain in specific gestational windows from weight gain in other periods of pregnancy.

### Latent class models

Latent class models are based on the assumption that exposures in a population, such as weight gain trajectories, are not comprised of a single homogenous group, but instead, consist of different heterogeneous subgroups (such as women with excess gain in early pregnancy vs women who gained similar total weight gain but in later pregnancy). These models estimate the number and size of latent classes (subgroups) in a given population based on observed data, and assign class membership to each woman. The latent classes can then be used as independent predictors in a model predicting adverse health outcomes, or, preferably, incorporated into a joint model with an outcome such as birthweight.^29^, ^30^

An important advantage of the latent class approach is that it avoids the simplifying assumptions needed to formally quantify or describe the broad variation observed in women's weight gain trajectories. However, the number of groups chosen by the model is data‐driven and may not necessarily correlate with clinically important outcomes. Furthermore, the extent to which findings could be translated into clinical and public health recommendations for pregnancy weight gain is unclear, as it is uncertain at what point in antenatal care group membership can be established.

### SITAR model

The SITAR model (Super Imposition by Translation And Rotation) is non‐linear mixed effects model that was developed to describe adolescent growth trajectories.^31^ The model is appealing because it enables complex weight trajectories to be summarised in terms of 3 parameters: the timing of growth (eg age at start of puberty), the velocity (eg the steepness of the growth curve), and total amount (eg final size achieved). Our team has recently applied the model to characterise pregnancy weight gain trajectories.^32^ We found that models aiming to fit all 3 parameters had challenges with convergence (potentially because the shape of the weight gain curve is less sigmoidal than adolescent growth curves), but reduced versions of the model that estimated 2 of the 3 parameters fit well. The 2 growth curve parameters describing each woman's weight gain trajectory can then be linked with maternal and child health outcomes. However, more work remains to better understand the value of the approach to the study of pregnancy weight gain.

## Accounting for the temporality of weight gain

Adverse outcomes that are diagnosed while pregnancy is ongoing (such as gestational diabetes, preeclampsia, or ultrasound‐diagnosed fetal growth restriction) may alter a woman's subsequent pregnancy weight gain. For example, women diagnosed with gestational diabetes are often referred for nutritional counselling, which can alter third‐trimester pregnancy weight gain.^33^, ^34^ In general, preeclampsia is clinically evident late in pregnancy, but many of the pathophysiological changes are present months earlier.^35^ The increased vascular permeability and decreased plasma oncotic pressure in preeclamptic pregnancies can cause significant oedema and rapid weight gain that would impact total gestational weight gain. If the goal is to estimate the association between pregnancy weight gain and risk of disease incidence, use of weight measurements taken after disease onset or diagnosis can introduce bias due to reverse causation. Instead, total weight gain *prior* to the onset of disease should be used to estimate potential causal effects.^1^ While data on the precise timing of disease onset can be challenging to obtain, researchers can nevertheless limit to weight gain prior to diagnosis or to the weight at the time of last normal values (eg last normal blood pressure).

## Isolating the causal effect of pregnancy weight gain

Research that aims to estimate potential causal effects of gestational weight gain on one or more adverse health outcomes must reduce bias due to confounding. Recent advances in epidemiology highlight the complexity of determining whether covariates meet the definition of a confounder.^36^ One way to address the complexity is through graphical representation of the causal relationships of the variables to one another, such as through the use of directed acyclic graphs (DAG).^37^, ^38^ Because the web of causal relationships among a typical set of covariates can be complex, researchers are encouraged to consider several step‐by‐step resources that offer a pragmatic solution to the problem.^39^ In particular, the role of past obstetrical history warrants careful consideration.^40^

**Figure 2:**
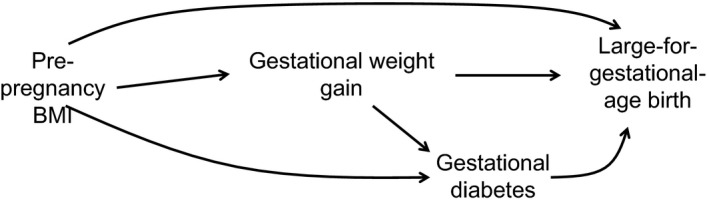
Directed acyclic graph of the link between pre‐pregnancy body mass index and large‐for‐gestational‐age birth.

Most researchers aim to estimate the overall association between gestational weight gain and an adverse birth outcome. To achieve this aim, variables that are on the causal pathway between gestational weight gain and adverse outcome (ie that are downstream from gestational weight gain) should not be included in the regression model. Doing so will remove the effect of gestational weight gain that is mediated through this intermediary variable. For example, as highlighted in a simplistic DAG in Figure 2, researchers interested in studying the effect of pregnancy weight gain on risk of large‐for‐gestational‐age birth should not adjust for gestational diabetes because it is both a downstream consequence of high pregnancy weight gain and a cause of large‐for‐gestational‐age birth.^41^ If gestational diabetes is included in the model, it will eliminate from the overall estimated effect of pregnancy weight gain the contribution mediated through that pathway.^36^ For a similar reason, when pre‐pregnancy BMI is the main exposure, adjustment for gestational weight gain is inappropriate. However, when pregnancy weight gain is the main exposure, adjustment for pre‐pregnancy BMI is necessary.

## Describing non‐linear associations

The risks of some adverse pregnancy outcomes may be increased with both low and high pregnancy weight gain. Examples of outcomes with a U‐ or J‐shaped association include preterm birth and infant mortality.^9^, ^42^ Researchers should verify the assumption of linearity when including pregnancy weight gain or weight gain *z*‐score as a linear term in a multivariable regression model. Methods for assessing linearity (eg regressing the outcome against quintiles of weight gain and examining the extent to which the resulting coefficients with 95% confidence intervals are compatible with a linear relationship) should be reported.

Strategies that retain weight gain as a continuous variable, such as fractional polynomials or restricted cubic splines, are preferable to categorising weight gain into quantiles or other groupings to reduce loss of information.^43^, ^44^ Interpretability of model coefficients can be facilitated by graphing risks of adverse outcomes across the weight gain continuum, and presenting measures of effect (eg risk ratios or absolute risk difference) with 95% confidence intervals for select values of weight gain. This can be easily implemented using the ‘margins’ command in Stata or R.

## Accounting for effect measure modification by pre‐pregnancy BMI

It is well established that the association between pregnancy weight gain and maternal and child health outcomes differs according to pre‐pregnancy BMI.^1^ To assess this effect modification, researchers should examine deviations from additive (rather than multiplicative) joint effects of BMI and gestational weight gain.^45^ Deviations from additivity identify subpopulations that may benefit from interventions and therefore are of greatest interest to policy makers. This effect measure modification by pre‐pregnancy BMI can be accounted for through several strategies.

The simplest strategy is to conduct analyses stratified by pre‐pregnancy BMI category. This approach produces results in a format that can more easily inform IOM guidelines, which are BMI category‐specific.^1^ However, the approach should not be used for studies where the aim is also to estimate the potential effects of pre‐pregnancy BMI, which cannot be estimated from stratified models. Additionally, accounting for deviations from additivity through stratification rather than multivariable regression is a less efficient use of the data from a statistical perspective,^46^ so may only be practical when large sample sizes are available.

An alternative approach is to specify a regression model that includes an interaction term between pre‐pregnancy BMI and pregnancy weight gain (in addition to main effects for each). As with any model that includes an interaction term, the effects of weight gain on adverse outcomes should then be presented at select values of pre‐pregnancy BMI (eg 18.5, 25, 30, 35 kg/m^2^). This can easily be implemented in standard statistical software packages using commands such as ‘margins’. Specifying BMI as a continuous variable (rather than categorised) also reduces the potential for residual confounding or incomplete accounting for the effect measure modification.^47^ Because pre‐pregnancy BMI often has a non‐linear relationship with outcomes of interest, strategies to account for non‐linear associations should be used (as discussed above).

## Assessing the contribution of systematic measurement error

Researchers must often rely on self‐reported measures of weight and height to calculate gestational weight gain or pre‐pregnancy BMI. On average, U.S. women tend to underestimate their weight and overestimate their height, but there is a wide range in the degree and direction of the misreporting.^48^ Although many researchers assert that this misclassification is non‐differential and will underestimate effects, this reasoning is often flawed.^49^ One large validation study found that the amount of measurement error introduced through self‐reported weight varied according to adverse outcome and categories of BMI and gestational weight gain.^50^

Analytic strategies that account for bias due to measurement error are recommended.^51^ Investigators should undertake validation studies to quantify the magnitude and direction of measurement error in self‐reported weights and/or heights within their own population. These data (or external validation data if appropriate) can then be applied to analytic strategies that account for measurement error, such as probabilistic bias analysis or Bayesian adjustment.^51^, ^52^, ^53^

## Drawing conclusions on optimal pregnancy weight ranges

An important motivation for pregnancy weight gain research is to inform guidelines on the recommended range of weight gain, such as those produced by the IOM. These guidelines are derived by synthesising available studies examining the link between pregnancy weight gain and a variety of adverse maternal and child health outcomes. Because low pregnancy weight gain increases the risk of some adverse outcomes (eg SGA birth and preterm birth), but decreases the risk of others (eg caesarean delivery, LGA birth, post‐partum weight retention), optimal weight gain ranges must be established through a balancing of risks.^1^ Researchers studying the association between weight gain and a single health outcome should therefore be cautious about drawing conclusions on the optimal range of gestational weight gain based solely on their results. Studies reporting on multiple adverse outcomes have greater potential to be interpreted in the light of optimal weight gain ranges, especially if they include many of the well‐established outcomes related to both high and low weight gain.^1^, ^54^, ^55^

## Conclusions

Observational studies of maternal obesity and suboptimal pregnancy weight gain can provide important insights into the role that these common and potentially modifiable risk factors play in many adverse pregnancy outcomes. However, such studies pose unique methodological challenges that, if not adequately accounted for, can bias study findings. Application of the principles outlined in this commentary will help to ensure that observational studies best isolate the contribution of maternal weight status to perinatal health from other potential influences, and help support public health recommendations for pregnancy weight gain that optimise the short‐ and long‐term health of mother and child.
